# Giant Popliteal Artery Aneurysm: Case Report and Review of the Literature

**DOI:** 10.1155/2014/780561

**Published:** 2014-08-21

**Authors:** Christos Verikokos, Georgios Karaolanis, Mikes Doulaptsis, Georgios Kouvelos, Aikaterini Kotzadimitriou, Viktoria-Varvara Palla, Christos Klonaris

**Affiliations:** ^1^2nd Department of Surgery, Vascular Surgery Unit, Laiko General Hospital, Medical School of Athens, Agiou Thoma 17, 11527 Athens, Greece; ^2^1st Department of Surgery, Vascular Surgery Unit, Laiko General Hospital, Medical School of Athens, Athens, Greece; ^3^Department of Gynecology, G. Genimatas General Hospital, Medical School of Athens, Athens, Greece

## Abstract

Popliteal artery aneurysms (PAAs) are rare in general population but represent the second most common peripheral arterial aneurysms following those located in the aortoiliac segment. They usually affect men over 60 years old with established cardiovascular disease caused by atherosclerosis. Other more unusual conditions such as trauma, congenital popliteal aneurysm, mycotic aneurysm, inflammatory arteritis, or popliteal entrapment are responsible. The authors report the first ever case of a male diagnosed with chronic renal failure with giant popliteal artery aneurysm. We have successfully resected the aneurysm and revascularized with synthetic graft.

## 1. Introduction

Popliteal artery aneurysms (PAAs) represent the second most common peripheral arterial aneurysms following those located in the aortoiliac segment. They account for approximately 70% of all peripheral aneurysms with an incidence estimated to be less than 0.1% [1]. They are more common in men over 60 years old with established cardiovascular disease and they are often associated with contralateral PAAs and abdominal aortic aneurysms [2]. PAAs are typically caused by atherosclerosis but they have also been observed in other more unusual conditions such as trauma, congenital popliteal aneurysm, mycotic aneurysm, inflammatory arteritis, or popliteal entrapment [3]. Huge PAAs, because of their anatomical position, size, and common complications, pose a therapeutic challenge for the vascular surgeon. We present a case of a male with a giant PAA treated surgically.

## 2. Case Report

A 63-year-old male was admitted to our department complaining of pain and swelling behind his left knee aggravating throughout the last month. The patient had a history of chronic renal failure under hemodialysis, hypertension, and diabetes mellitus and was a heavy smoker. He had undergone saphenous vein ablation on the right leg due to significant venous insufficiency. There was no history of any trauma or local/systemic infection.

On examination, a palpable pulsatile mass in the upper popliteal fossa was revealed. Left distal pulses were weak, while the ankle brachial index (ABI) for the left and right limb was 0.8 and >1, respectively.

**Figure 1:**
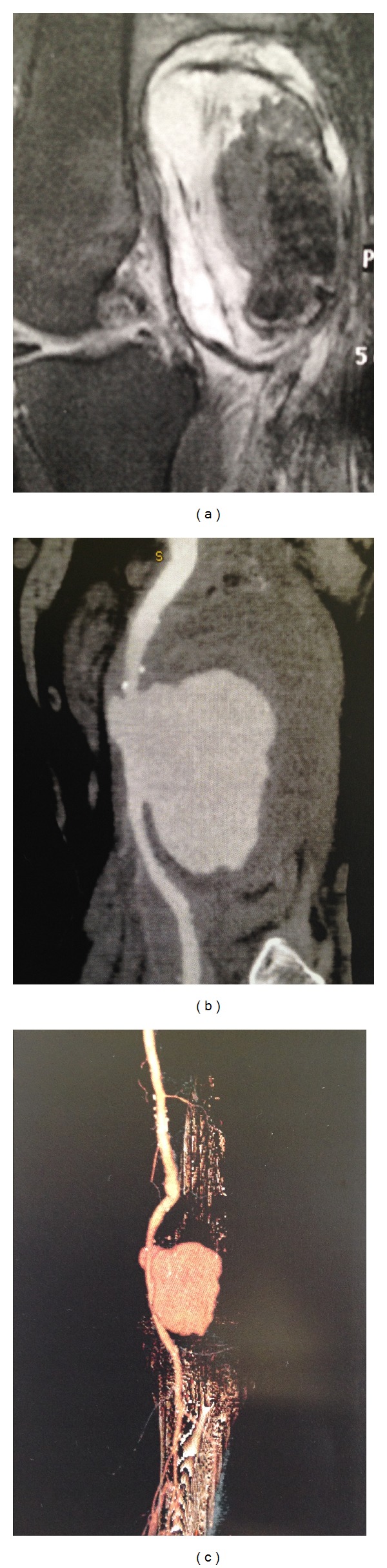
(a) Magnetic resonance imaging (MRI) showing the huge popliteal artery aneurysm. (b) Computed tomography angiography (CTA) depicting the huge aneurysm with significant thrombus inside the aneurysm sac. (c) 3D reconstruction of CTA showing the aneurysm with the inflow and outflow vessels.

Magnetic resonance imaging (MRI) revealed the presence of a popliteal mass with a cephalocaudal diameter of 11 cm (Figure 1). Color duplex ultrasonography demonstrated a huge PAA with an uneven echogenic collection inside and a thrombosed left popliteal vein, along with diminished flow of the tibial arteries. Computed tomography angiogram (CTA) depicted a multilobulated mass (5.6 × 5.6 × 14.3 cm) extending from the midthigh to popliteal fossa, with the presence of thrombus inside (Figure 1).

“After discussing with the patient his therapeutic options for an open or an endovascular procedure, he decided for an open approach, as a more durable solution, despite the greater perioperative risk.”

## 3. Procedure

**Figure 2:**
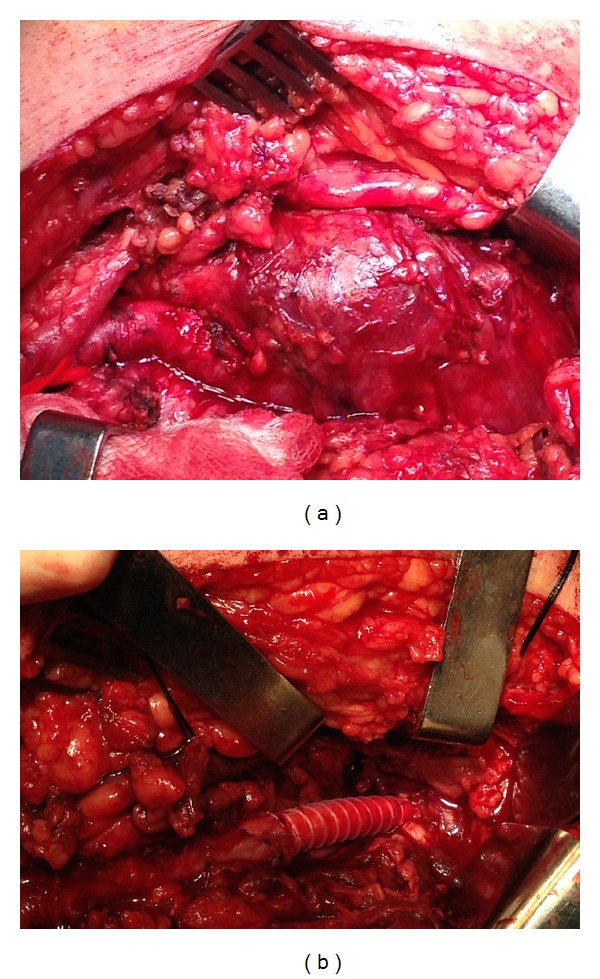
(a) Giant popliteal artery aneurysm intraoperatively. (b) Revascularization with polytetrafluoroethylene (PTFE) graft.

With medial approach, the distal superficial femoral artery and the distal popliteal artery were exposed to allow proximal and distal arterial control (Figure 2). The aneurysm sac was then opened and both the thrombus and the wall were removed and sent for histological examination. A bypass grafting was performed from the distal femoral artery to the distal popliteal artery above the knee with the use of a polytetrafluoroethylene (PTFE) graft in an end-to-side fashion (Figure 2). The patient had an uneventful postoperative course and was discharged in good general condition with pulsatile pedal pulses. At the 6-month follow-up, the patient was well and suffered no complications. Duplex scan showed excellent graft patency with no other complications.

## 4. Discussion

PAA is the most common peripheral aneurysm presenting bilaterally in 50% and coexisting with abdominal aortic aneurysm (AAA) in 50% of cases. Although 80% are asymptomatic at the time of diagnosis [4], they become symptomatic at a rate of 14% per year [5]. In the literature, five cases of giant PAA treated have been reported: one case of bilateral popliteal artery entrapment syndrome [6], one of a posttraumatic popliteal artery pseudoaneurysm, occurring during a soccer game [7], and another one in a young child of congenital aetiology [8]. There is also another case of a ruptured giant popliteal aneurysm occurring in an 84-year-old male who presented with a multilobulated mass on the left leg and a large mass on the right leg. In this case, the authors proceeded to the evacuation of the multilobulated mass of the left leg without surgical revascularization because of the absence of symptoms and the presence of an adequate collateral blood flow. On the other limb, ligation and bypass with nonreversed greater saphenous vein were performed [9]. Additionally, a case of an 88-year-old woman with a PAA of 11 cm has been reported. The patient was unfit for open surgery because of her advanced age and the concomitant comorbidities, so an endovascular approach was elected. The results were satisfactory and the woman has an uneventful postoperative course [10].

Large aneurysms are at risk of serious complications such as arterial embolization, thrombosis, and, less commonly, rupture. Although the indications for repair of PAAs are not well defined, a diameter greater than 2 cm to 3 cm, especially for aneurysms with a significant thrombus load or with chronic distal tibial artery embolic occlusion, is an acceptable indication for intervention [11, 12]. Patients with severe claudication, resting pain, and tissue loss are considered for elective repair, as are patients who have symptoms of local compression. Indications for asymptomatic patients and for those with mild-to-moderate claudication are less well defined. Recently, the approach to repair these aneurysms has become a subject of debate, particularly with the increased utilization of endovascular techniques [13]. Different studies comparing both techniques have been published. Tsilimparis et al. in a retrospective analysis presented the data of studies published in the past 25 years and came to the conclusion that open surgical repair remains the gold standard but the endovascular repair is being performed more commonly with acceptable results [14]. Furthermore, Galiñanes et al. in a retrospective analysis of 2962 patients showed that the rates of reinterventions during 30 and 90 days after initial procedure were considerably greater after endovascular procedure than after open surgery [15]. In addition, open approach appeared to convey greater durability and greater cost benefit than those of endovascular approach [15, 16]. An important point was that this approach was suggested for patients of a good health status. They may benefit from the durability of an open repair and avoid the higher reintervention rates associated with an endovascular approach [15]. Pulli et al. analysed early and follow-up results of the treatment of PAA performed with open surgical repair or with endovascular exclusion with endografts, involving seven Italian vascular centers. Similarly, they reported the same results for the two procedures [11]. In our case, based on patient's age and the compromised distal run-off, we decided to proceed with an open surgical approach to achieve a more durable result and avoid the risk for reintervention. Moreover, due to the huge size of the aneurysm followed by mechanical compression of adjacent structures, the surgical exploration with hematoma evacuation and arterial repair was the most appropriate approach. Nevertheless, in young patients, limitation of endovascular technique consists of the inability to obtain cultures or biopsies in order to diagnose infection or congenital abnormality.

In conclusion, giant PAA is a rare entity with very limited number of cases published so far. Compression issues and patient's characteristics should be meticulously considered in treatment decision. Open surgical approach seems to comprise a durable and adequate therapeutic strategy in such cases.
